# Microglia Loss and Astrocyte Activation Cause Dynamic Changes in Hippocampal [^18^F]DPA-714 Uptake in Mouse Models of Depression

**DOI:** 10.3389/fncel.2022.802192

**Published:** 2022-02-18

**Authors:** Jiamei Guo, Tian Qiu, Lixia Wang, Lei Shi, Ming Ai, Zhu Xia, Zhiping Peng, Anhai Zheng, Xiao Li, Li Kuang

**Affiliations:** ^1^Department of Psychiatry, The First Affiliated Hospital of Chongqing Medical University, Chongqing, China; ^2^Department of Nuclear Medicine, The First Affiliated Hospital of Chongqing Medical University, Chongqing, China; ^3^College of Basic Medicine, Chongqing Medical University, Chongqing, China

**Keywords:** depression, microglia, chronic unpredictable stress, [^18^F]DPA-714 PET, astrocytes

## Abstract

Major depression is a serious and chronic mental illness. However, its etiology is poorly understood. Although glial cells have been increasingly implicated in the pathogenesis of depression, the specific role of microglia and astrocytes in stress-induced depression remains unclear. Translocator protein (TSPO) has long been considered a marker of neuroinflammation and microglial activation. However, this protein is also present on astrocytes. Thus, it is necessary to explore the relationships between TSPO, microglia, and astrocytes in the context of depression. In this study, C57BL/6J male mice were subjected to chronic unpredictable stress (CUS) for 5 weeks. Subsequently, sucrose preference and tail suspension tests (TSTs) were performed to assess anhedonia and despair in these mice. [^18^F]DPA-714 positron emission tomography (PET) was adopted to dynamically assess the changes in glial cells before and 2, 4, or 5 weeks after CUS exposure. The numbers of TSPO^+^ cells, ionized calcium-binding adaptor molecule (Iba)-1^+^ microglial cells, TSPO^+^/Iba-1^+^ cells, glial fibrillary acidic protein (GFAP)^+^ astrocytes, TSPO^+^/GFAP^+^ cells, and TUNEL-stained microglia were quantified using immunofluorescence staining. Real-time PCR was used to evaluate interleukin *(IL)-1*β, *IL-4*, and *IL-18* expression in the hippocampus. We observed that hippocampal [^18^F]DPA-714 uptake significantly increased after 2 weeks of CUS. However, the signal significantly decreased after 5 weeks of CUS. CUS significantly reduced the number of Iba-1^+^, TSPO^+^, and TSPO^+^/Iba-1^+^ cells in the hippocampus, especially in the CA1 and dentate gyrus (DG) subregions. However, this intervention increased the number of GFAP^+^ astrocytes in the CA2/CA3 subregions of the hippocampus. In addition, microglial apoptosis in the early stage of CUS appeared to be involved in microglia loss. Further, the expression of pro-inflammatory cytokines (*IL-1*β and *IL-18)* was significantly decreased after CUS. In contrast, the expression of the anti-inflammatory cytokine *IL-4* was significantly increased after 2 weeks of CUS. These results suggested that the CUS-induced dynamic changes in hippocampal [^18^F]DPA-714 uptake and several cytokines may be due to combined microglial and astrocyte action. These findings provide a theoretical reference for the future clinical applications of TSPO PET.

## Introduction

Major depression is a serious and chronic mental illness, affecting approximately 350 million people worldwide (Malhi and Mann, 2018). Antidepressants are effective in only 60–70% of cases, and the treatment outcomes in patients with depression remain unsatisfactory (Rush et al., 2006). Such outcomes severely impact patient quality of life and create a heavy social burden (GBD 2016 Disease and Injury Incidence and Prevalence Collaborators, 2017). Unfortunately, the etiology of depression and relevant antidepressant mechanisms are poorly understood (Otte et al., 2016).

Previous studies on depression have largely focused on neuronal mechanisms and neural loops (Fang et al., 2021; Malberg et al., 2021). However, in recent years, growing evidence has shown that glial cells are also involved in the pathogenesis of depression (Rial et al., 2015; Czéh and Nagy, 2018; Durkee et al., 2021) and could hold the key for its diagnosis and prevention.

Among the many pathogenic hypotheses of depression, the inflammatory hypothesis has attracted substantial attention (Pfau et al., 2018; Stepanichev et al., 2018). Microglia, resident macrophages of the central nervous system (CNS), play an important role in maintaining innate immunity in the CNS (Mrdjen et al., 2018; Greenhalgh et al., 2020). Therefore, most studies have focused on the role of microglia in neuroinflammation. Previous reports suggest that microglia remain in a resting state until they are activated in the presence of a threat. However, recent studies have shown that although the somas of microglia are stationary under conditions of homeostasis, their processes are dynamic and continuously survey the surrounding environment (Madry et al., 2018). Typically, microglial activation follows two types of patterns. Under the pro-inflammatory pattern, the secretion of pro-inflammatory cytokines [interleukin (IL)-1, IL-6, tumor necrosis factor (TNF), etc.] is promoted, while under the anti-inflammatory pattern, anti-inflammatory cytokines, such as IL-4 and IL-10, are secreted (Orihuela et al., 2016). Several studies suggest that microglial activation in the hippocampus mediates the depressive behaviors induced by chronic unpredictable stress (CUS) and chronic mild stress (CMS) (Wang et al., 2018; Du Preez et al., 2021). Recent studies show that dynamic transitions across microglial states could play a critical role in the pathophysiology of depression (Steiner et al., 2011; Kreisel et al., 2014; Tang et al., 2018; Zhang et al., 2018). However, the pro- vs. anti-inflammatory dichotomy in microglia is now being challenged. Several studies have demonstrated that microglial responses to injury are neither completely good nor bad but instead represent a continuum of states (Cherry et al., 2014; Loane and Kumar, 2016). As such, the impairment of normal microglial structure and function contributes to the etiology of depression. Astrocytes, as the primary homeostatic cells of the CNS, also play a significant role in inflammation and depression (Rowitch and Kriegstein, 2010). Similar to microglia, astrocytes get activated in the presence of threats against the CNS (Guttenplan and Liddelow, 2019). Activated astrocytes can also promote the secretion of anti-inflammatory cytokines (Liddelow and Barres, 2017; Xie et al., 2020). Conversely, pro-inflammatory cytokines, especially IL-1β, can cause astrocytes to secrete neurotrophic factors critical for neuron survival (Suzumura et al., 2006). Moreover, chronic activation causes astrocytes to produce high levels of chemokines (Gimsa et al., 2013). Furthermore, hippocampal astrocytes mediate the depressive behavior induced by chronic stress (Hisaoka-Nakashima et al., 2020; Du Preez et al., 2021). Taken together, these findings indicate that dynamic changes in glia and their interactions contribute to the inflammatory processes involved in depression.

Translocator protein (TSPO; 18 kDa) is an important protein. It is located on the outer mitochondrial membranes of cells in peripheral tissues and the CNS (Lacapere et al., 2020). TSPO is widely recognized as an indicator of microglial activation and neuroinflammation (Jain et al., 2020). Normally, TSPO expression in the brain is very low. However, this protein is significantly upregulated in cases of craniocerebral trauma, multiple sclerosis, and encephalitis (Setiawan et al., 2018; Kang et al., 2021). The rapid development of positron emission tomography (PET) technology and TSPO ligands has enabled improved monitoring of TSPO levels in different organs *in vivo* (Kopschina et al., 2019; Zinnhardt et al., 2020). PET with radiolabeled ligands specific for TSPO is widely used to assess aberrant neuroinflammation in patients with psychiatric or neurological diseases (Zinnhardt et al., 2020). For example, clinical studies have shown that the total distribution of TSPO is increased in patients with depression (Setiawan et al., 2015). Moreover, this increase is more significant in patients with suicidal ideation or a long disease duration (Setiawan et al., 2018). Therefore, PET-detected TSPO levels are considered potential markers for precision therapy. So far, TSPO PET has primarily been used to examine microglial activation in small animals *in vivo* (Wang et al., 2018; Kopschina et al., 2019). However, dynamic changes in TSPO under conditions of chronic stress have not been examined. In some inflammatory diseases, TSPO can reflect microglial activation and neuroinflammatory levels *in vivo*. However, TSPO is also located on astrocytes, which are closely associated with inflammation (Notter et al., 2021). Understanding the cellular localization of TSPO in different glial cells in non-inflammatory diseases such as depression could be clinically useful. In addition, the dynamic monitoring of TSPO levels *in vivo* could help elucidate how TSPO, microglia, and astrocytes interact during the pathogenesis of depression. Such information could provide new ideas for mechanistic research on depression.

The hippocampus is a key brain region responsible for advanced functions such as learning, memory, and emotion. The hippocampus sends nerve afferents to the frontal cortex, amygdala, and other emotion-related brain areas (Battaglia et al., 2011). Cumulative evidence shows that hippocampal structure and function affect the pathogenesis of depression (Khan et al., 2019). Moreover, as mentioned earlier, hippocampal microglia and astrocytes contribute to the pathophysiology of depression (Du Preez et al., 2021). The total TSPO distribution volume appears to be elevated in the hippocampus in depression patients (Li et al., 2018). Therefore, the present study mainly focused on hippocampal glia. Various radioisotope-labeled TSPO ligands have been developed and utilized for autoradiography or PET scans *in vivo* (Plavén-Sigray et al., 2020). One such ligand is [^18^F]DPA-714, which has superior target specificity. This ligand also shows a higher binding affinity and signal-to-noise ratio than other TSPO radioligands (Hamelin et al., 2018). Furthermore, the physical properties of F-18 make it very suitable for *in vivo* PET imaging (Jacobson et al., 2015).

Thus, the broad aim of this study was to dynamically evaluate hippocampal TSPO expression, the number of hippocampal microglia and astrocytes, and the TSPO expression in glial cells in a CUS-induced mouse model of depression. Further, we examined how these variables differ across the subregions of the hippocampus [CA1, CA2/3, and dentate gyrus (DG) regions]. For these assessments, we used a combination of [^18^F]DPA-714 PET imaging *in vivo*, real-time PCR, and immunofluorescence assays. Our results revealed dynamic changes in TSPO levels and localization in microglia and astrocytes during stress, thereby providing a theoretical basis for studying glial changes during the onset of depression.

## Materials and Methods

### Animals

Six-week-old male C57BL/6J mice were obtained from the Laboratory Animal Center of Chongqing Medical University (Chongqing, PR China). All the animals were housed in groups of five per cage under controlled environmental conditions (12-h light/dark cycle; lights on from 08:00 to 20:00; 22 ± 2°C ambient temperature; 55 ± 10% relative humidity). The animals had free access to standard laboratory feed and water. All experimental procedures and animal care protocols complied with the National Institutes of Health Guide for the Care and Use of Laboratory Animals and the Laboratory Animal Guidelines of Chongqing Medical University. The protocol was approved by the Animal Research Committee of the First Affiliated Hospital of Chongqing Medical University.

### Chronic Unpredictable Stress Intervention

**FIGURE 1:**
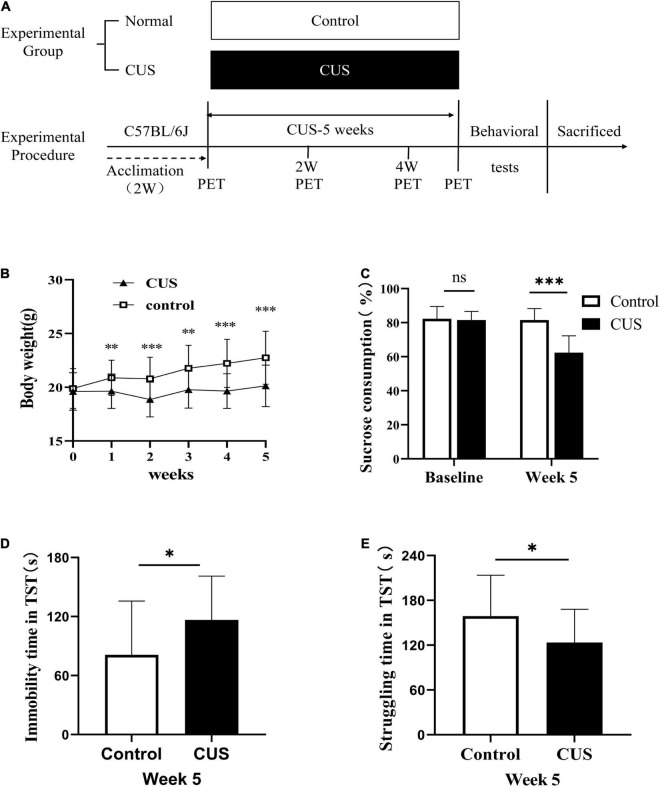
Experimental procedures and results of behavioral experiments. **(A)** Experimental procedures. **(B)** Body weight changes during the 5-week study period (mean ± *SD*, *n* = 18–20/control group, *n* = 28–60/CUS group, unpaired *t*-test at each time-point). **(C)** Sucrose preference after 5 weeks of CUS intervention (mean + *SD*, *n* = 18–20/control group, *n* = 28–55/CUS group, unpaired *t*-test). **(D)** Immobility duration in the TST after 5 weeks of CUS intervention (mean ± *SD*, *n* = 18/control group, *n* = 28/CUS group, unpaired *t*-test). **(E)** Struggling time in the TST after 5 weeks of CUS intervention (mean ± *SD*, *n* = 18/control group, *n* = 28/CUS group, unpaired *t*-test). * indicates *P* < 0.05; ^**^ indicates *P* < 0.01; ^***^ indicates *P* < 0.001. ns indicates *P* > 0.05. CUS, chronic unpredictable stress; TST, tail suspension test.

Animals were habituated to the housing conditions for 2 weeks. Next, the animals were randomly divided into the CUS group (stressed mice, *n* = 60) and normal control group (control mice, *n* = 20) (Figure 1A). The CUS group was further divided into two subgroups (CUS-2W group: mice subjected to 2 weeks of CUS; CUS-5W group: mice subjected to 5 weeks of CUS). Each mouse in the CUS group experienced a group of stressors known to induce depressive behavior in rodents. The mice were randomly exposed to two of the following stressors daily for 5 weeks (Fang et al., 2021): food and/or water deprivation, wet bedding, cage tilting, light changes (on/off), cage shaking, light flashes, electric shock, restraint, and cold stress. Mice in the control group were housed under normal conditions. Body weights and sucrose preference were evaluated once every week. The tail suspension test (TST) was performed after 5 weeks of CUS.

### Behavioral Tests

#### Sucrose Preference Test

The Sucrose Preference Test (SPT) was used to evaluate anhedonia (Naranjo et al., 2020). The SPT was carried out every weekend at 9:00 a.m. For this test, the mice were put into individual cages. They were allowed to drink from two bottles, one containing fresh water and the other containing a 1% sucrose solution, and left undisturbed for 24 h. The position of the bottle containing the sucrose solution (left vs. right) was randomly chosen. Sucrose preference was computed based on the percentage of sucrose consumption out of the total liquid consumption.

#### Tail Suspension Test

The TST was performed according to previously published protocols (Li et al., 2021). Briefly, the mice in the two groups were individually suspended 50 cm above the floor for 6 min. The mice were suspended using adhesive tape placed approximately 1 cm from the tip of the tail. The duration of immobility was recorded during the last 4 min by an investigator blinded to group allocation. Mice were considered immobile only when they hung passively and were completely motionless. Any mouse that climbed its tail was excluded from the experimental analysis.

### Double Immunofluorescence

Ionized calcium-binding adaptor molecule-1 (Iba-1) and glial fibrillary acidic protein (GFAP) are considered markers for microglia and reactive astrocytes, respectively (Golabchi et al., 2019). Mice were sacrificed immediately after the behavioral tests were completed. Immunofluorescence assays were performed as described previously (Ren et al., 2016). The mice were deeply anesthetized with pentobarbital sodium and perfused transcardially with 4% paraformaldehyde in 0.01 M phosphate buffer. The brain tissue was removed and fixed in 4% paraformaldehyde and stored at 4°C for immunofluorescence analysis. The brains were dehydrated, dipped in paraffin and embedded, and cut into 3–5-μm sections. After deparaffinization, the sections were heated in a microwave oven for 25 min in EDTA antigen retrieval buffer (pH 8). Subsequently, the tissue sections were immersed in 3% H_2_O_2_ and incubated in goat serum in 3% BSA (Servicebio, Wuhan, China) for 30 min. The tissue sections were then incubated with a mixture of anti-TSPO (1:2,000, Abcam, United Kingdom) and anti-Iba-1 (1:1,000, Novus, United States) or anti-GFAP antibodies (1:2,000, Abcam, United Kingdom) at 4°C overnight. After washing in PBS, sections were incubated with fluorescein isothiocyanate (FITC)-conjugated goat anti-rabbit IgG (1:100, Servicebio, Wuhan, China) and tetramethylrhodamine isothiocyanate (TRITC)-conjugated goat anti-mouse IgG (1:100, Servicebio, Wuhan, China) for 90 min at 37°C. The sections were washed in PBS and incubated with a DAPI solution for 10 min. Then, the sections were mounted on slides and coverslipped. Fluorescence was assessed using laser scanning confocal microscopy (Leica Microsystems Heidelberg GmbH, Germany) with an Olympus SP2 inverted microscope (Olympus) equipped with a Fluoview FVX confocal scanhead. The number of hippocampal cells positive for Iba-1, TSPO, and GFAP and the number of hippocampal cells showing the co-localization of these proteins (TSPO^+^/Iba-1^+^ and TSPO^+^/GFAP^+^) were quantified. Microglial process length and the area of the microglial soma were quantified using ImageJ (software, National Institutes of Health). Apoptosis was detected using a terminal deoxynucleotidyl transferase nick-end labeling (TUNEL) kit, according to the manufacturer’s protocol (Beyotime Biotechnology, Shanghai, China).

### [^18^F]DPA-714 PET

[^18^F]DPA-714 PET scans were performed using a nano-Scan PET/CT system (Mediso Ltd., Budapest, Hungary). Efforts were made to use the same mice for PET assessments at baseline and 2, 4, and 5 weeks after CUS intervention. For the procedure, the mice were anesthetized with isoflurane, and [^18^F]DPA-714 was injected via the tail vein. Mice were anesthetized 40 min after tracer injection and placed in a prone position in the center of the scanner. Whole brain computed tomography (CT) scans and 20-min static PET scans were obtained. All list-mode data were reconstructed using the OSEM 2D algorithm (frames, 4 × 30, 5 × 150, 6 × 450 s) without attenuation correction. The reconstructed PET images were manually aligned with the MRI template atlas of the mouse brain using the image analysis software PMOD (3.7, PMOD Technologies, Zurich, Switzerland).^1^ Hippocampi were selected as the volumes of interest. The uptake of [^18^F]DPA-714 was presented as a standardized uptake value (SUV) and measured 0–20 min after injection.

### Quantitative Real-Time PCR

Mice were sacrificed using cervical dislocation, and fresh brain tissue samples were obtained. The tissue samples were immediately placed in liquid nitrogen and stored at –80°C for real-time PCR. Total RNA was extracted from the hippocampus using the total RNA tissue extraction kit (Tiangen Biotech, Beijing, China) according to the manufacturer’s instructions. The RNA concentration was measured using a NanoDrop 2000 system (Thermo Fisher Scientific). The RNA was reverse transcribed into cDNA using a reverse transcription kit (Servicebio, Wuhan, China). Real-time RT-PCR was performed using the ABI Prism 7500 real-time PCR System. Relative gene expression was normalized based on *GAPDH* expression using the 2–^ΔΔ^ Ct method. Primer sequences were as follows:

TSPO (sense, GCAGAAACCCTCTTGGCATC; antisense, AG CGTCCTCTGTGAAACCTCC); IL-1β(sense, GCATCCAGCTT CAAATCTCGC; anti-sense, TGTTCATCTCGGAGCCTGT AGTG); IL-4 (sense,TGTCATCCTGCTCTTCTTTCTCG; anti-sense, TTTGGCACATCCATCTCCGT); IL-18 (sense, TGAAGTAAGAGGACTGGCTGTGA; anti-sense, TTGGCAA GCAAGAAAGTGTCC); GAPDH (sense, CCTCGTCCCGTA GACAAAATG; antisense, TGAGGTCAATGAAGGGGTCGT);

## Results

### Chronic Unpredictable Stress Induced Depressive Behavior in C57BL/6J Mice

To elucidate the role of glial cells in depression, mice were subjected to CUS, a classic model of depression (Fang et al., 2021). At baseline, sucrose preference (Figure 1C, 82.25 ± 7.25 vs. 81.51 ± 5.13, *p* = 0.618) and body weight (Figure 1B, 19.88 ± 1.86 vs. 19.61 ± 1.75, *p* = 0.567) were similar between the control and CUS groups. However, in the second week of CUS, the body weight of mice in the CUS group was observed to be significantly lower than that of mice in the control group (Figure 1B, 20.88 ± 1.64 vs. 19.63 ± 1.6, *p* = 0.006). As expected, after 5 weeks of CUS, mice in the CUS group showed a significantly lower preference for sucrose than mice in the control group (Figure 1C, 81.51 ± 6.82 vs. 62.4 ± 9.85, *p* < 0.001). Moreover, these mice showed a higher duration of immobility (Figure 1D, 81.05 ± 54.73 vs. 116.58 ± 44.59, *P* = 0.028) and a corresponding lower duration of struggle in the TST (Figure 1E, 158.95 ± 64.73 vs. 123.42 ± 44.59, *P* = 0.028).

### Chronic Unpredictable Stress Caused Dynamic Changes in [^18^F]DPA-714 Uptake in the Hippocampus

**FIGURE 2:**
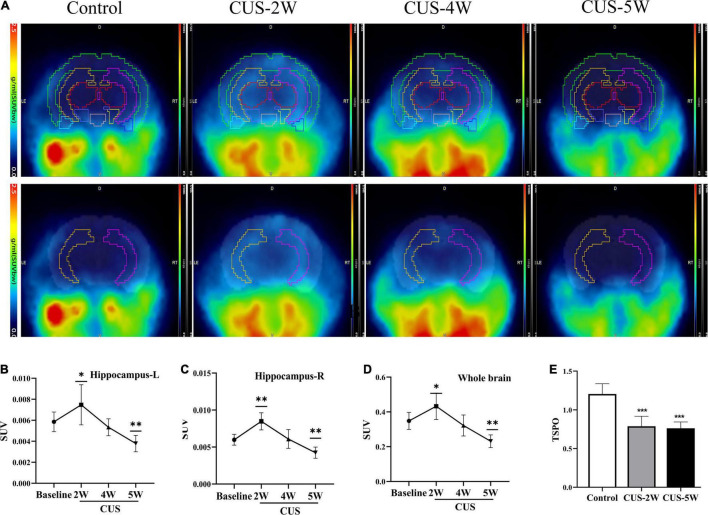
CUS induces dynamic changes in hippocampal [^18^F]DPA-714 signals. **(A)** Dynamic changes of [^18^F]DPA-714 SUV in the whole brain and hippocampus during 5-weekCUS intervention. The results represent normalized MRI images derived from individual images. **(B,C)** [^18^F]DPA-714 SUVs in the hippocampus at different time-points during CUS intervention (mean ± *SD*, *n* = 4–6/group, one-way ANOVA test); **(B)** represents the left hippocampus and **(C)** represents the right hippocampus. **(D)** [^18^F]DPA-714 SUVs in the whole brain at different time-points during CUS intervention (mean ± *SD*, *n* = 4–6/group, one-way ANOVA test). **(E)** Expression of *TSPO* (RT-PCR) in the hippocampus in the control and CUS groups (meant ± *SD*, *n* = 5/group, one-way ANOVA test) * indicates *P* < 0.05; ^**^ indicates *P* < 0.01; *** indicates *P* < 0.001. CUS, chronic unpredictable stress; SUV, standard uptake value. CUS-2W: mice experienced 2 weeks of CUS; CUS-4W: mice experienced 4 weeks of CUS; CUS-5W: mice experienced 5 weeks of CUS.

[^18^F]DPA-714, a new specific TSPO radioligand, allows the quantification of TSPO levels *in vivo* via PET imaging (Barca et al., 2021). In our study, six male C57BL/6J mice underwent [^18^F]DPA-714 PET before and after 2, 4, or 5 weeks of exposure to CUS (Figure 2A). As illustrated in Figures 2B,C, the SUV of [^18^F]DPA-714 in the hippocampus (Figure 2B, left hippocampus, 0.0059 ± 0.0009 vs. 0.0075 ± 0.0019, *P* = 0.034; Figure 2C, right hippocampus, 0.006 ± 0.0007 vs. 0.0085 + 0.0012, *P* = 0.001) increased after 2 weeks of CUS. However, this value significantly decreased after 5 weeks of CUS compared with baseline values (Figure 2B, left hippocampus, 0.0059 ± 0.0009 vs. 0.0075 ± 0.0019, *P* = 0.034; Figure 2C, right hippocampus, 0.006 ± 0.0007 vs. 0.0085 + 0.0012, *P* = 0.001). Moreover, there was no significant difference between these values at baseline and after 4 weeks of CUS (Figure 2B, left hippocampus, 0.0059 ± 0.0009 vs. 0.0053 ± 0.0008, *P* = 0.424; Figure 2C, right hippocampus, 0.006 ± 0.0007 vs. 0.0061 ± 0.0013, *P* = 0.863). Further, similar results were observed in whole-brain analyses (Figure 2D). The results illustrated that short-term CUS significantly increases the SUV of [^18^F]DPA-714 in the hippocampus. However, long-term CUS significantly decreases these levels. We then analyzed the RNA levels of *TSPO* using real-time PCR. We observed that hippocampal *TSPO* levels were significantly lower in stressed mice than in control mice (Figure 2E, 1.204 ± 0.133 vs. 0.788 ± 0.129 vs. 0.762 ± 0.082, *P*_Control, CUS–2W_ < 0.001, *P*_Control, CUS–5W_ < 0.001). The results showed that CUS reduced hippocampal *TSPO* levels *ex vivo.*

### Chronic Unpredictable Stress Decreased the Number of TSPO^+^ Cells and Microglia in the Hippocampus

**FIGURE 3:**
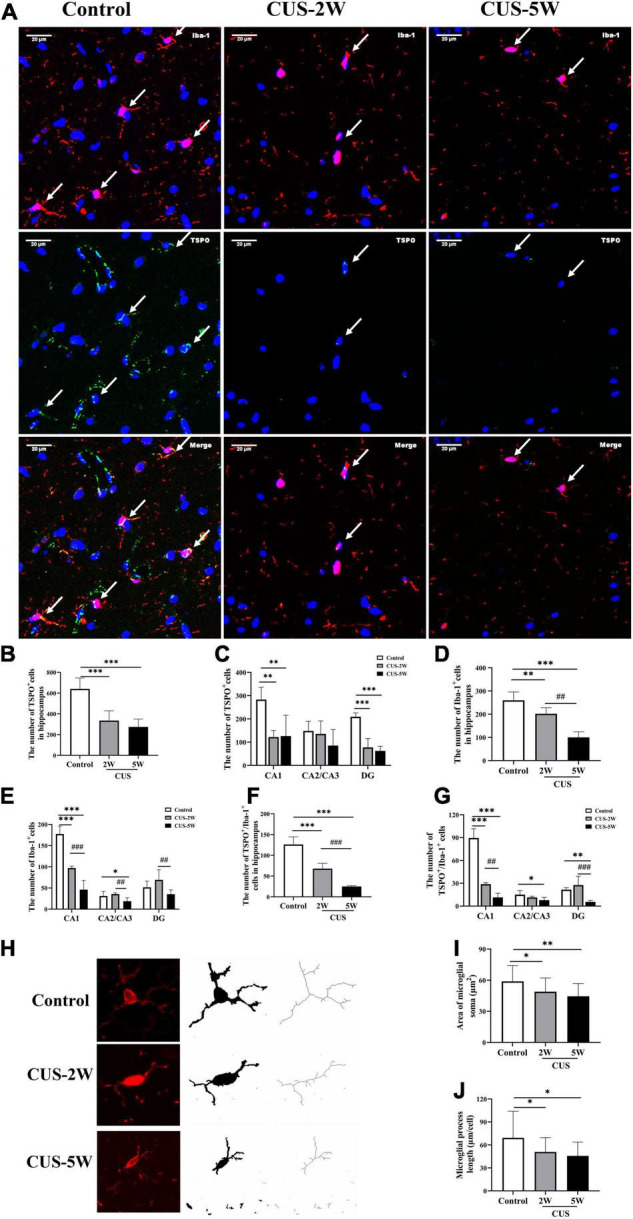
Effects of CUS on TSPO^+^ cells, microglia, and microglial morphology in the hippocampus. **(A)** Representative images of immunofluorescence staining with anti-TSPO and anti-Iba-1 antibodies. White arrows indicate positive cells. **(B,C)** Quantification of TSPO^+^ cells in the hippocampus and its three subregions (meant ± *SD*, *n* = 5/group, one-way ANOVA test). **(D,E)** Quantification of Iba-1^+^ cells in the hippocampus and its three subregions (meant ± *SD*, *n* = 5/group, one-way ANOVA test). **(F,G)** Quantification of TSPO^+^/Iba-1^+^ cells in the hippocampus and its three subregions (meant ± *SD*, *n* = 5/group, one-way ANOVA test). **(H)** Representative images of Microglia under immunofluorescence and cytoskeleton. **(I)** Area of Microglial soma in the control and CUS groups (meant ± *SD*, *n* = 20/group, one-way ANOVA test). **(J)** Microglial process length in the control and CUS groups (meant ± *SD*, *n* = 16–17/group, one-way ANOVA test). * indicates *P* < 0.05; ^**^ indicates *P* < 0.01 and ^***^ indicates *P* < 0.001 for comparison between the stress and control groups. ^##^ indicates *P* < 0.01 and ^###^ indicates *P* < 0.001 for comparison between the CUS-2W and CUS-5W groups. CUS-2W group, mice experienced 2 weeks of CUS; CUS-5W group, mice experienced 5 weeks of CUS.

Representative images of immunofluorescence staining for TSPO and Iba-1 are shown in Figure 3A. The numbers of TSPO^+^ cells in the hippocampus and its three subregions in the stressed and control groups are shown in Figures 3B,C. We observed that the numbers of TSPO^+^ cells in the whole hippocampus (Figure 3B, 640.2 ± 106.5 vs. 335.1 ± 94.8 vs. 274.3 ± 76.1, *P*_Control, CUS–2W_ < 0.001, *P*_Control, CUS–5W_ < 0.001), CA1 subregion (Figure 3C, 283 ± 53.1 vs. 97 ± 4.5 vs. 46 ± 21.9, *P*_Control, CUS–2W_ = 0.002, *P*_Control, CUS–5W_ = 0.002) and DG subregion (Figure 3C, 51.6 ± 14.5 vs. 69.2 ± 23.8 vs. 35.4 ± 10.2, *P*_Control, CUS–2W_ < 0.001, *P*_Control, CUS–5W_ < 0.001) were significantly decreased after 2 and 5 weeks of CUS. This indicated that CUS decreased the expression of TSPO in the hippocampus, especially in the CA1 and DG subregions.

Further, the numbers of Iba-1^+^ microglial cells in the hippocampus (Figure 3D, 259.87 ± 35.96 vs. 201.87 ± 25.99 vs. 100.2 ± 23.95, *P*_Control, CUS–2W_ = 0.008, *P*_Control, CUS–5W_ < 0.001, *P*_CUS–2W, CUS–5W_ < 0.001) and CA1 subregion (Figure 3E, 177.4 ± 18.62 vs. 97 ± 4.53 vs. 46 ± 21.94, *P*_Control, CUS–2W_ < 0.001, *P*_Control, CUS–5W_ < 0.001, *P*_CUS–2W, CUS–5W_ < 0.001) were found to be significantly lower in mice subjected to 2 and 5 weeks of CUS than in control mice. This decrease appeared to be time-dependent. The number of microglial cells in the CA2/CA3 subregion was also significantly decreased after 5 weeks of CUS (Figure 3E, 31 ± 10.68 vs. 35.6 ± 2.79 vs. 18.8 ± 8.17, *P*_Control, CUS–5W_ = 0.032, *P*_CUS–2W, CUS–5W_ = 0.006). However, only the number of microglial cells in the DG was significantly different between the two CUS subgroups (Figure 3E, 51.6 ± 14.5 vs. 69.2 ± 23.78 vs. 35.4 ± 10.21, *P*_CUS–2W, CUS–5W_ = 0.009). These results indicated that after short-term CUS (2 weeks), microglia loss occurred in the hippocampus.

The number of TSPO^+^/Iba-1^+^ cells in the hippocampus was calculated to evaluate TSPO expression in microglia. Overall, changes in TSPO expression in hippocampal microglia were largely consistent with the microglial changes occurring during stress-induced depression. The numbers of Iba-1^+^/TSPO^+^ cells in the hippocampus (Figure 3F, 259.87 ± 35.96 vs. 201.87 ± 25.99 vs. 100.2 ± 23.95, *P*_Control, CUS–2W_ < 0.001, *P*_Control, CUS–5W_ < 0.001, *P*_CUS–2W, CUS–5W_ < 0.001) and the CA1 subregion (Figure 3G, 89.6 ± 12.12 vs. 28.8 ± 2.05 vs. 11.4 ± 5.41 *P* < 0.05) were significantly lower in mice subjected to 2 and 5 weeks of CUS than in control mice. This decrease also appeared to be time-dependent. Further, there was a significant decrease in the number of TSPO^+^/Iba-1^+^ cells in CA2/CA3 (Figure 3G, 15 ± 5.43 vs. 11 ± 1.67 vs. 7.8 ± 3.63, *P*_Control, CUS–5W_ = 0.013) and DG (Figure 3G, 21.6 ± 2.61 vs. 27.67 ± 11.55 vs. 5.6 ± 2.07, *P*_Control, CUS–5W_ = 0.003) subregions after 5 weeks of CUS intervention.

### Chronic Unpredictable Stress Altered Microglial Morphology and Increased the Number of Apoptotic Microglia

The morphology of microglia reflects their function and state of activation (Abiega et al., 2016; Mrdjen et al., 2018). We explored the dynamic effects of CUS on microglial morphology in the hippocampus. Microglial process length and soma area were quantified using ImageJ software. Morphological data were collected from fluorescence images by the AnalyzeSkeleton (2D/3D) plugin and converted to representative binary and skeletonized images for quantitative measurements in ImageJ (Figure 3H). As shown in Figures 3I,J, microglial morphology was altered in the hippocampus following CUS, with smaller microglial soma (Figure 3I, 58.89 ± 15.24 vs. 48.93 ± 13.25 vs. 44.48 ± 12.3, *P*_Control, CUS–2W_ = 0.025, *P*_Control, CUS–5W_ = 0.001), shorter process length (Figure 3J, 69.26 ± 34.77 vs. 50.8 ± 18.86 vs. 45.77 ± 18.03, *P*_Control, CUS–2W_ = 0.039, *P*_Control, CUS–5W_ = 0.011) and fewer process branches. These results suggested that CUS intervention caused the changes in morphology of microglia.

**FIGURE 4:**
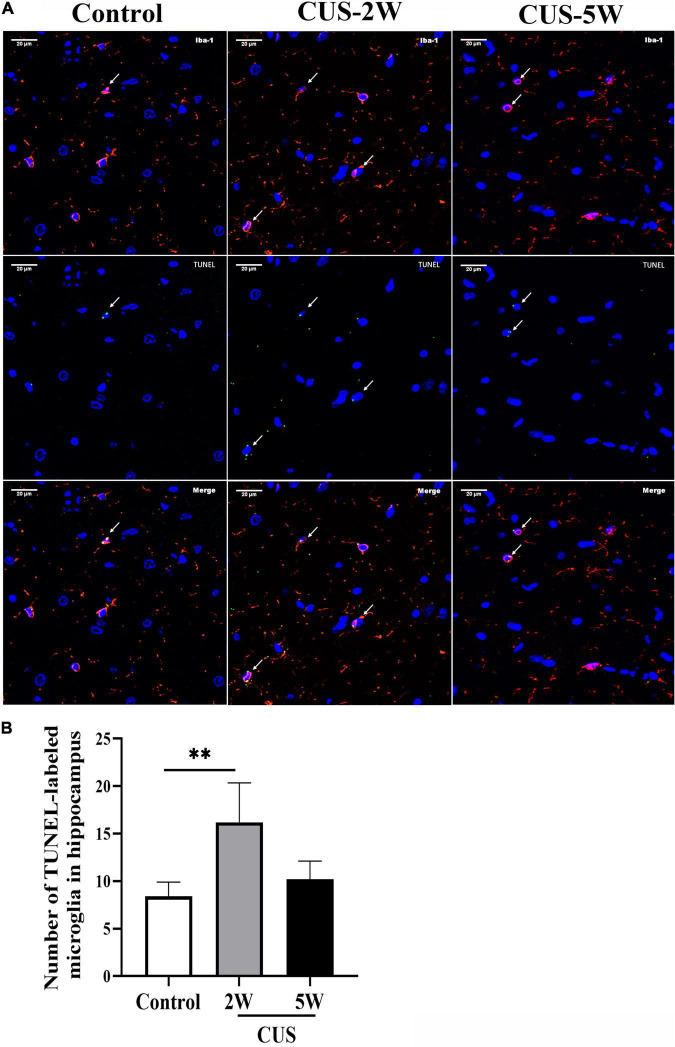
Effects of CUS intervention on the number of apoptotic microglia. White arrows indicate positive cells. **(A)** Representative images of TUNEL-stained apoptotic microglia at low and high magnification. **(B)** Quantification analysis of the number of TUNEL-stained microglia in the hippocampus (meant ± *SD*, *n* = 5/group, one-way ANOVA test). ^**^ indicates *P* < 0.01. CUS-2W group, mice experienced 2 weeks of CUS; CUS-5W group, mice experienced 5 weeks of CUS.

To further evaluate the effects of CUS intervention on microglia in the hippocampus, we evaluated the TUNEL-stained apoptotic microglia at 2 and 5weeks post-CUS intervention. Representative images of TUNEL-stained apoptotic microglia are shown in Figure 4A. The results showed the numbers of TUNEL-stained microglia markedly increased after 2 weeks (not after 5 weeks) CUS intervention (Figure 4B, 8.4 ± 1.52 vs. 16.2 ± 4.15 vs. 10.2 ± 1.92, *P*_Control, CUS–2W_ = 0.001, *P*_Control, CUS–5W_ = 0.326). This suggested that microglial apoptosis in the hippocampus may contribute to the loss of microglia observed in CUS-induced mouse models of depression.

### Chronic Unpredictable Stress Increased the Number of Astrocytes in the Hippocampus

**FIGURE 5:**
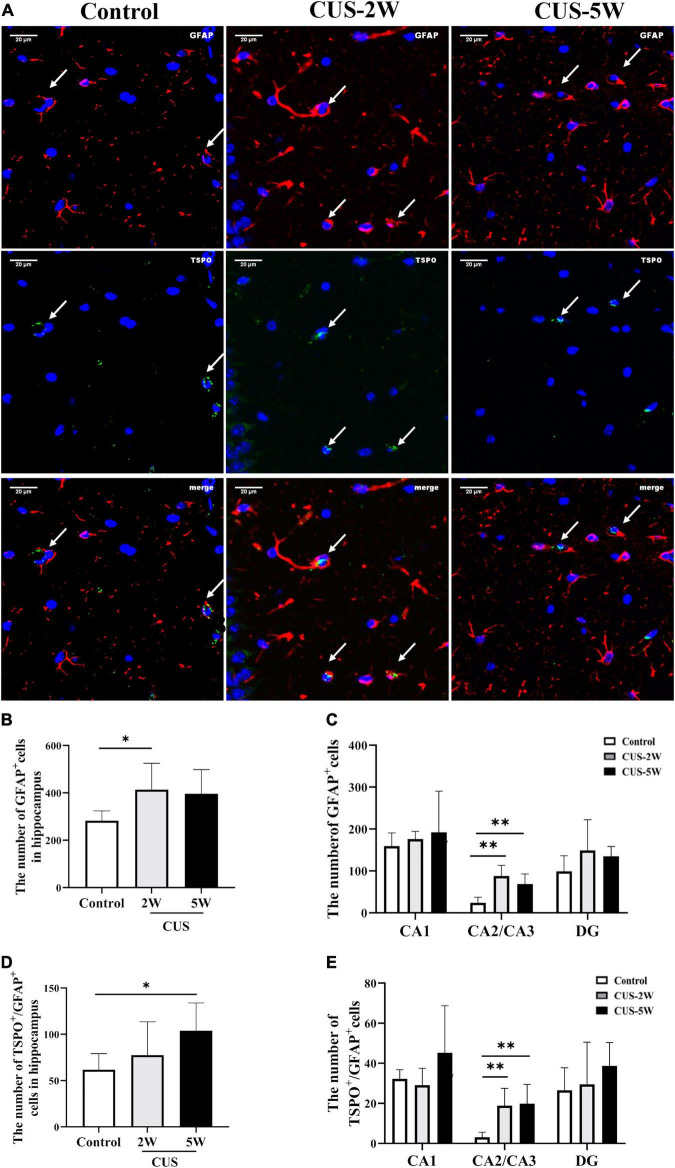
Effects of CUS on GFAP^+^ astrocytes. **(A)** Representative images of immunofluorescence staining with anti-TSPO and anti-GFAP antibodies. White arrows indicate positive cells. **(B,C)** Quantification of GFAP^+^ cells in the hippocampus and its three subregions (meant ± *SD*, *n* = 5/group, one-way ANOVA test). **(D,E)** Quantification of TSPO^+^/GFAP^+^ cells in the hippocampus and its three subregions (meant ± *SD*, *n* = 5/group, one-way ANOVA test). * indicates *P* < 0.05; ^**^ indicates *P* < 0.01. CUS-2W group, mice experienced 2 weeks of CUS; CUS-5W group, mice experienced 5 weeks of CUS.

CUS intervention initially caused an increase in the hippocampal [^18^F]DPA-714 signal. However, prolonged CUS resulted in a decreased [^18^F]DPA-714 signal. Nevertheless, it was interesting to note that the number of Iba-1^+^ cells and Iba-1^+^/TSPO^+^ cells showed a continuous time-dependent decrease after CUS. Therefore, we hypothesized that CUS could increase the number of other glial cells expressing TSPO, such as astrocytes. Representative images of GFAP and TSPO immunostaining are shown in Figure 5A. The mean total number of GFAP^+^ and TSPO^+^/GFAP^+^ cells in the hippocampus s and its three subregions is shown in Figures 5B–E. We observed that the number of GFAP^+^ cells was significantly increased in the hippocampus (Figure 5B, 282.73 ± 41.51 vs. 413.67 ± 111.64 vs. 396 ± 102.55, *P*_Control, CUS–2W_ = 0.042) and in the CA2/CA3 subregion (Figure 5C, 24.07 ± 12.98 vs. 88.07 ± 25.56 vs. 68.8 ± 24.07, *P*_Control, CUS–2W_ = 0.001) after 2 weeks of CUS. These results indicated that short-term CUS led to a significant increase in astrocytes. However, there was no significant difference between the control group and stressed group after 5 weeks of CUS (Figure 5B, *P*_Control, CUS–5W_ = 0.072).

TSPO^+^/GFAP^+^ co-localization was used to observe TSPO expression in hippocampal astrocytes. The number of TSPO^+^/GFAP^+^ cells in the hippocampus was significantly increased after 5 weeks of CUS (Figure 4D, 61.8 ± 17.34 vs. 77.6 ± 35.93 vs. 103.8 ± 30.18, *P*_Control, CUS–5W_ = 0.042). However, only a trend toward an increase was observed after 2 weeks of CUS. Interestingly, the number of TSPO^+^/GFAP^+^ cells in the CA2/CA3 subregion was significantly increased after both 2 and 5 weeks of CUS (Figure 4E, 3.13 ± 2.47 vs. 18.93 ± 8.55 vs. 19.87 ± 9.56, *P*_Control, CUS–2W_ = 0.006, *P*_Control, CUS–5W_ = 0.004).

### Chronic Unpredictable Stress Induced Dynamic Changes in Cytokine Expression (IL-1β, IL-18, and IL-4)

**FIGURE 6:**
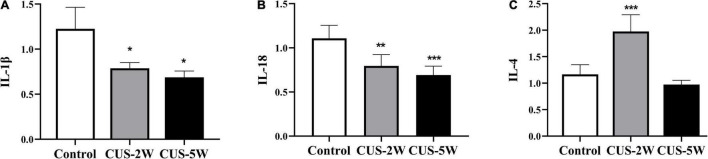
Effects of CUS on pro-inflammatory and anti-inflammatory cytokines. **(A)** Expression of *IL-1*β (RT-PCR) in the hippocampus in the control and CUS groups (meant ± *SD*, *n* = 5/group, one-way ANOVA test). **(B)** Expression of *IL-18* (RT-PCR) in the hippocampus in the control and CUS groups (meant ± *SD*, *n* = 5/group, one-way ANOVA test). **(C)** Expression of *IL-4* (RT-PCR) in the hippocampus in the control and CUS groups (meant ± *SD*, *n* = 5/group, one-way ANOVA test). * indicates *P* < 0.05; ** indicates *P* < 0.01; and *** indicates *P* < 0.001. CUS-2W group, mice experienced 2 weeks of CUS; CUS-5W group, mice experienced 5 weeks of CUS.

We further used RT-PCR to detect the levels of several cytokines closely associated with glia. The results showed that the levels of *IL-1*β (Figure 6A, 1.226 ± 0.238 vs. 0.788 ± 0.062 vs. 0.688 ± 0.07, *P*_Control, CUS–2W_ = 0.028, *P*_Control, CUS–5W_ = 0.013) and *IL-18* (Figure 6B, 1.108 ± 0.148 vs. 0.796 ± 0.128 vs. 0.692 ± 0.101, *P*_Control, CUS–2W_ = 0.002, *P*_Control, CUS–5W_ < 0.001), which are associated with the NLRP3 (nucleotide-binding oligomerization domain-like receptor family, pyrin domain containing 3) inflammasome, were significantly decreased after 2 and 5 weeks of CUS. These trends were consistent with microglia loss after CUS intervention. Furthermore, we observed a significant increase in the levels of the anti-inflammatory cytokine *IL-4* after 2 weeks of CUS (Figure 6C, 1.166 ± 0.183 vs. 1.976 ± 0.316 vs. 0.974 ± 0.796,*P*_Control, CUS–2W_ < 0.001, *P*_Control, CUS–5W_ = 0.185). However, these levels showed a downward trend after 5 weeks of CUS.

## Discussion

CUS is one of the most commonly used modeling approaches for depressive behavior (Fang et al., 2021). In our study, the immobility time in the TST was used to assess despair, and sucrose preference in the SPT was used to assess anhedonia. We found that CUS significantly reduced sucrose preference and increased the immobility time. These results were consistent with those of previous studies, indicating that the CUS-induced depression model was successfully established (Lin et al., 2021).

[^18^F]DPA-714 PET has been used in preclinical (Barca et al., 2021) and clinical research (Setiawan et al., 2018) to quantify TSPO levels *in vivo*. Our results confirmed that [^18^F]DPA-714 successfully bound to hippocampal cells in mice. We found that CUS caused [^18^F]DPA-714 uptake to first increase at 2 weeks and then decrease after 5 weeks of CUS. Therefore, the time-dependent changes in SUVs showed an “inverted V”-shaped graph. The values after 4 weeks of stress were similar to those at baseline. Interestingly, this result contradicted previous findings and was unexpected, as we predicted that [^18^F]DPA-714 signals would gradually increase with an increase in CUS duration. Wang et al. (2018) used [^18^F]DPA-714 PET to detect microglial activation in adult rats after CMS exposure and only observed an increase in hippocampal [^18^F]DPA-714 signals. However, there were two primary differences between our study and Wang et al. (2018). First, in our study, [^18^F]DPA-714 SUVs were detected at four time points and reflected dynamic [^18^F]DPA-714 SUV variations during the course of CUS. In contrast, Wang et al. (2018) compared differences in [^18^F]DPA-714 signals only after 12 weeks of CMS. Therefore, one advantage offered by our study was the dynamic monitoring of TSPO changes *in vivo* during CUS-induced depression. Second, while our study used a 5-week CUS intervention to induce depressive behavior in juvenile mice, in the study by Wang et al. (2018) adult rats were randomly subjected to one mild stressor daily over a 12-week period. Hence, the animal model and species also differed between the studies. Stimulus intensity is stronger in CUS than in CMS, but the stress duration is shorter (Gururajan et al., 2019). Studies have shown that strong and persistent stimulation can cause cell atrophy and death. Moreover, predictable mild stress has been reported to improve depression-like behavior (Dang et al., 2019). Therefore, the two models may simulate different stages or types of depression, which could also partly account for differences in the results. We speculate that in the early stage of stress, glial cells are activated and start to proliferate. In contrast, glial atrophy and loss occur in the later stages of stress. In future studies, we will identify the changes in glial cell proliferation and apoptosis during stress-induced depression.

The gold standard for evaluating CNS abnormalities is post-mortem examination; however, these examinations have two remarkable limitations. First, the effects of external factors cannot be ruled out in this system. Second, longitudinal assessments of disease progression cannot be performed. In contrast, *in vivo* neuroimaging studies, such as PET, allow the observation of CNS abnormalities during the clinical course of a disease (Kopschina et al., 2019). Most previous studies equate TSPO signals with microglial activation or neuroinflammation (Wang et al., 2018; Jain et al., 2020). However, it should be noted that TSPO is also significantly up-regulated in activated astrocytes (Notter et al., 2018). Our study confirmed that TSPO was expressed in both activated microglia and astrocytes in the mouse hippocampus. We also observed a progressive decrease in the number of hippocampal microglia and Iba-1^+^/TSPO^+^ cells following CUS intervention. The CA1 subregion was the first and most significantly affected hippocampal subregion. Hence, the variations in the number of microglia and Iba-1^+^/TSPO^+^ cells were not consistent with the increase in TSPO radiotracer binding after 2 weeks of CUS. Interestingly, after CUS intervention, the number of GFAP^+^ astrocytes and TSPO^+^/GFAP^+^ cells was significantly increased in the hippocampus, especially in the CA2/CA3 subregions. These results indicated that short-term CUS causes an increase in astrocytes. Further, the increased TSPO expression in astrocytes could partly explain the increase in [^18^F]DPA-714 SUVs after the 2-week CUS intervention. Hence, the dynamic changes in hippocampal SUVs during CUS may partially be attributed to the dynamic changes associated with microglia loss and astrocyte activation. The increase in [^18^F]DPA-714 SUVs after the 2-week CUS intervention may result from astrocytes activation. Moreover, the decrease after 5 weeks may primarily be caused by microglia loss. Based on this finding, we speculate that the use of [^18^F]DPA-714 PET to dynamically monitor the state of glia may be more beneficial for guiding clinical treatment.

The hippocampus has well-organized structures, and its subregions have specific functional roles (Andersen et al., 2006). Studies have found that the CA1 region plays an important role in the pathogenesis of depression. It has been reported that acute elevations in corticosterone levels induce synaptic plasticity in the CA1 region, and chronic stress reduces synaptic plasticity in both the DG and CA1 regions (Song et al., 2018). Further, a clinical study showed that alterations in the left CA1 region could be a potential marker of depression (Roddy et al., 2019). Microglia display a region-specific density and morphology. Their function also differs across different brain regions (Silvin and Ginhoux, 2018). Therefore, we speculate that the changes in TSPO signals and the number of microglia in the CA1 region are likely to be early indicators of depression. In line with this, Kreisel et al. (2014) first revealed that the loss of microglia in the hippocampus contributes to CUS-induced depression in animals. Subsequently, Tong et al. (2017) found similar results across three different models of depression induced by different types of chronic stress. These studies further suggested that apoptosis may contribute to the loss of microglia after initial microglial activation. In our study, the CUS group showed a smaller microglial soma area and shorter process length than the control group. Further, our results indicated that increased microglial apoptosis in the early stages of stress may lead to a subsequent loss of microglia, which was largely consistent with previous results (Kreisel et al., 2014). Microglia and Neurotrophic substances that they secrete play an important role in neural plasticity and Neurogenesis (Hughes and Appel, 2020). It would be valuable to reveal the mechanism underlying the microglia loss observed in CUS-induced depression. Caspases proteases are key proteins mediating apoptosis (Shalini et al., 2015). Caspase-3,-7, and -6 have been found to serve as effector caspases in apoptosis (Galluzzi et al., 2018). Recently, caspase-1 was also found to drive cells toward apoptosis instead of pyroptosis in the absence of GSDMD (Tsuchiya et al., 2019). In addition, the initial overactivation of microglia and cellular nutritional disorders may attribute to microglia loss (Deng et al., 2020). Furthermore, it has also been reported that the mechanisms of microglia loss may involve P2 × 4 receptors (Vázquez-Villoldo et al., 2014), deoxyglucose-mediated ATP depletion (Vilalta and Brown, 2014), and an increase in cellular lipid accumulation (Zhuang et al., 2021). However, studies focusing on the mechanisms of microglia loss remain limited. We aim to explore the mechanisms underlying stress-induced microglia loss during depression in future studies.

Lipopolysaccharide (LPS) and macrophage colony-stimulating factor (M-CSF)—which are known to induce microglial activation and thereby produce depression-like behavior—have been found to attenuate the CUS-induced behavioral abnormalities via reversing CUS-induced reductions in the hippocampal microglia numbers (Tong et al., 2017). Moreover, pretreatment with minocycline, which inhibits the initial phase of microglial activation (2–3 days of stress), was found to recapitulate the effects of LPS and M-CSF. Hence, preventing or reversing overactivation-induced microglia loss could help in the treatment of depression (Kreisel et al., 2014). We found that microglia decreased in the hippocampus after 2 weeks of CUS. This time-point was not examined in Kreisel et al. (2014) study, and our findings may thus provide a reference for selecting the treatment window. We speculate that anti-inflammatory drugs used prior to microglia loss (within 1 week of stress) may prevent microglial reductions during the future course of the disease, thus reversing depression. The efficacy of anti-inflammatory drugs against depression has long been debated (Hang et al., 2021; Kloiber et al., 2021), and this controversy may partly be associated with alterations in the state of hippocampal microglia during depression. The use of anti-inflammatory drugs may improve depressive symptoms only when microglia are activated. However, when depression is caused by a decrease in microglia, pro-inflammatory drugs may be effective in reducing symptoms through microglial activation (Tong et al., 2017). Therefore, we propose that the internal levels of inflammation should be evaluated before administering anti- or pro-inflammatory treatment. In the present study, we found that TSPO PET could be a valid method for estimating the status of glia *in vivo*. Our results motivate the use of TSPO PET in clinical and preclinical research for identifying the optimal treatment window and developing treatment plans for depression.

Astrocytes are the most abundant glial cells in the CNS (Rowitch and Kriegstein, 2010). These cells are required for synaptic function and neurotransmitter regulation—processes crucial for neuronal survival, growth, and differentiation as well as synaptic plasticity (Rial et al., 2015; Naranjo et al., 2020; Du Preez et al., 2021). Our results showed that short-term CUS intervention (2 weeks) led to significant astrocyte activation and an increase in the number of astrocytes, mainly in the CA2/CA3 subregions. Strong evidence shows that a decrease in hippocampal astrocytes (Eldomiaty et al., 2020), GFAP expression, GFAP-immunoreactive astrocytes (Yao et al., 2021), and astrocyte density (Virmani et al., 2021) is related to depression. However, studies also show that astrocyte activation may be involved in the pathophysiology of depression, supporting our findings. For example, a recent preclinical study reported that 6 weeks of CUS followed by another 6 weeks of social isolation induced an increase in the number of GFAP^+^ cells in the DG (Du Preez et al., 2021). Moreover, Michel et al. (2021) found that the level of GFAP was significantly higher in patients with unipolar depression than in mentally healthy controls with idiopathic intracranial hypertension. Hence, both the loss and increase of astrocytes may be responsible for stress-induced depressive behavior. However, these phenomenon may occur at different stages of depression. To our knowledge, our study is the first to dynamically evaluate the effect of short-term and long-term CUS on astrocytes in the hippocampus and its subregions. Based on our results, we speculate that the number of astrocytes may increase in early stage depression but decrease after a prolonged disease duration. The inhibition of astrocyte activation in the early stages of stress could reverse astrocyte loss in the later stages. These findings could provide new approaches for treating depression. It has been reported that the area fraction of GFAP-immunoreactivity in the hippocampal CA2/CA3 subregions is negatively correlated with the duration of depression in suicide victims (Cobb et al., 2016). Hence, in future studies, we will attempt to further elucidate the mechanisms of astrocyte activation in the CA2/CA3 subregions after short-term CUS. Further, we will verify whether the inhibition of astrocyte activation in the early stages can alleviate the depression-like behavior induced by chronic stress.

Microglia are known to play a critical role in inflammation (Mao et al., 2018; Stepanichev et al., 2018). Our study showed that the levels of the pro-inflammatory cytokines IL-1 and IL-18, which are associated with NLRP3, were decreased after CUS intervention. These changes were consistent with the microglia loss observed after CUS. We speculate that the decrease in pro-inflammatory cytokines (IL-1 and IL-18) could be involved in microglia loss. Elevated levels of pro-inflammatory cytokines released due to microglial activation have been observed in multiple brain regions in animals with depressive behaviors (Adinolfi et al., 2018; Pfau et al., 2018). However, it has also been reported that the decline in glial cells in some brain regions constitutes an important aspect of depression pathogenesis (Cotter et al., 2001; Bowley et al., 2002). Post-mortem single-cell analyses have revealed no evidence of inflammatory molecule induction in the microglia in patients with major depressive disorder (Böttcher et al., 2020; Snijders et al., 2020). Therefore, low levels of neuroinflammation may also promote depressive symptoms in patients. Interestingly, we found that the anti-inflammatory cytokine IL-4 was significantly elevated after 2 weeks of CUS. The alterations in the cytokine IL-4 could not be explained by microglia loss alone. However, the changes were consistent with astrocyte activation. Activated astrocytes can promote the secretion of the anti-inflammatory cytokine IL-4 (Lu et al., 2021; Zhang et al., 2021). Hence, the increase in IL-4 after the 2-week CUS intervention may be associated with astrocyte activation. Therefore, we speculate that different glia play different roles in the inflammatory cascade at different stages of stress-induced depression. Nevertheless, how CUS triggers an increase in the anti-inflammatory cytokine IL-4 and a decrease in pro-inflammatory cytokines remains to be elucidated.

## Conclusion

In summary, depression is a complex and highly heterogeneous disease. TSPO PET is a valid method for evaluating the status of glia *in vivo*. CUS intervention can cause microglia loss in the CA1 and CA2/CA3 regions of the hippocampus and also result in astrocyte activation in the CA2/CA3 region. Therefore, both hippocampal microglia and astrocytes appear to be involved in the pathogenesis of depression. More importantly, the changes in TSPO PET signals during CUS intervention may result from dynamic alterations in microglia and astrocytes in different hippocampal subregions. The changes in several pro- and anti-inflammatory cytokines may also be related to the variations in microglia and astrocytes. Additional *in vivo* and *ex vivo* experiments that combine and dynamically assess the changes in various hippocampal cells are required to further explore the etiology of depression and develop new treatments for this condition.

## Data Availability Statement

The original contributions presented in the study are included in the article/supplementary material, further inquiries can be directed to the corresponding author/s.

## Ethics Statement

The animal study was reviewed and approved by the Animal Research Committee of the First Affiliated Hospital of Chongqing Medical University.

## Author Contributions

JG, TQ, LW, and LS performed the main experiments. MA, ZX, and ZP performed the PET/CT scan. AZ and XL participate in building depression model. JG wrote the manuscript. LK initiated and supported the study. All authors discussed the manuscript.

## Conflict of Interest

The authors declare that the research was conducted in the absence of any commercial or financial relationships that could be construed as a potential conflict of interest.

## Publisher’s Note

All claims expressed in this article are solely those of the authors and do not necessarily represent those of their affiliated organizations, or those of the publisher, the editors and the reviewers. Any product that may be evaluated in this article, or claim that may be made by its manufacturer, is not guaranteed or endorsed by the publisher.

## Footnotes

1
www.pmod.com
